# Supplementary material for: Antimicrobial Use in Animals in Timor-Leste Based on Veterinary Antimicrobial Imports between 2016 and 2019
Source: Antibiotics (Basel). 2021 Apr 12;10(4):426. doi: 10.3390/antibiotics10040426 (PMC8070255; doi:10.3390/antibiotics10040426)
Supplement: Supplementary file 1 [file antibiotics-10-00426-s001.zip › Supp Material/Table S1 Biomass (final).docx]

**Table S1: Animal biomass for buffalo, cattle, chicken, goats, horses, pigs and sheep in Timor-Leste between 2016 and 2019.**

| Species | Year | Animal population^1^ | Animals slaughtered^1^ | Meat harvested^1^ (tonnes) | Carcass weight^2^ (kg) | Liveweight^3^  (kg) | Animal biomass^4^ (tonnes) |
| --- | --- | --- | --- | --- | --- | --- | --- |
| Buffalo | 2016 | 139059 | 7356 | 736.0 | 100.1 | 175.5 | 24409.6 |
|  | 2017 | 124186 | 6539 | 654.0 | 100.0 | 175.5 | 21790.3 |
|  | 2018 | 124824 | 6542 | 654.0 | 100.0 | 175.4 | 21892.3 |
|  | 2019 | 126066 | 6576 | 658.0 | 100.1 | 175.5 | 22130.3 |
| Cattle | 2016 | 229087 | 12893 | 1328.0 | 103.0 | 180.7 | 41397.1 |
|  | 2017 | 205663 | 11293 | 1163.0 | 103.0 | 180.7 | 37157.9 |
|  | 2018 | 209218 | 11201 | 1154.0 | 103.0 | 180.7 | 37815.8 |
|  | 2019 | 213235 | 11124 | 1146.0 | 103.0 | 180.7 | 38539.6 |
| Chicken | 2016 | 932000 | 1153000 | 922.0 | 0.8 | 1.1 | 1317.1 |
|  | 2017 | 950000 | 1178000 | 943.0 | 0.8 | 1.1 | 1347.1 |
|  | 2018 | 941000 | 1162000 | 929.0 | 0.8 | 1.1 | 1327.1 |
|  | 2019 | 904000 | 1118000 | 894.0 | 0.8 | 1.1 | 1277.1 |
| Goats | 2016 | 136608 | 38743 | 388.0 | 10.0 | 21.3 | 9134.0 |
|  | 2017 | 95810 | 27040 | 271.0 | 10.0 | 21.3 | 6410.3 |
|  | 2018 | 74870 | 21027 | 210.0 | 10.0 | 21.2 | 5010.7 |
|  | 2019 | 66504 | 18586 | 186.0 | 10.0 | 21.3 | 4454.2 |
| Horse | 2016 | 52032 | - | - | - | 165.5 | 8611.3 |
|  | 2017 | 52998 | - | - | - | 165.5 | 8771.2 |
|  | 2018 | 51251 | - | - | - | 165.5 | 8482.0 |
|  | 2019 | 50777 | - | - | - | 165.5 | 8403.6 |
| Pigs | 2016 | 416078 | 286893 | 11476.0 | 40.0 | 51.3 | 23700.1 |
|  | 2017 | 388319 | 270209 | 10808.0 | 40.0 | 51.3 | 22244.1 |
|  | 2018 | 390875 | 274460 | 10978.0 | 40.0 | 51.3 | 22517.3 |
|  | 2019 | 393869 | 279053 | 11162.0 | 40.0 | 51.3 | 22817.8 |
| Sheep | 2016 | 29587 | 4781 | 48.0 | 10.0 | 21.4 | 2082.1 |
|  | 2017 | 43334 | 7080 | 71.0 | 10.0 | 21.3 | 3047.1 |
|  | 2018 | 41848 | 6913 | 69.0 | 10.0 | 21.2 | 2939.8 |
|  | 2019 | 42593 | 7112 | 71.0 | 10.0 | 21.2 | 2989.9 |

^1^ Data was obtained from the FAOSTAT database. Data on animals slaughtered and meat harvested was not available for horses in Timor-Leste.

^2^ Carcass weight was calculated by dividing “Meat harvested (tonnes)” by “Animals Slaughtered”.

^3^ Liveweight was determined by dividing “Carcass weight (kg)” by a conversion coefficient. Conversion coefficients were species specific: 0.54 for buffalo and cattle, 0.7 for chicken, 0.47 for goats and sheep, and 0.78 for pigs. The liveweight for horses was determined based on the population-wide mean bodyweight of a local breed of horse in Timor-Leste.

^4^ Animal biomass was estimated based on OIE methodology [34] except for bovine biomass where it was calculated by multiplying “Animal population” by “Liveweight (kg)”.
