# Supplementary material for: Antimicrobial Use in Animals in Timor-Leste Based on Veterinary Antimicrobial Imports between 2016 and 2019
Source: Antibiotics (Basel). 2021 Apr 12;10(4):426. doi: 10.3390/antibiotics10040426 (PMC8070255; doi:10.3390/antibiotics10040426)
Supplement: Supplementary file 1 [file antibiotics-10-00426-s001.zip › Supp Material/Table S2 Route of administration (final).docx]

Table S2: Import weight of various veterinary antimicrobial classes by route of administration between 2016 and 2019.

|  |  | Kilogram of active ingredient (%) | | | | | Kilogram for each route of administration from 2016 to 2019 (%) |
| --- | --- | --- | --- | --- | --- | --- | --- |
| Route of administration | Antimicrobial class | 2016 | 2017 | 2018 | 2019 | Total from 2016 to 2019 |  |
| Topical | Aminoglycosides | 0 | 2.5x10^-4^ (0.28) | 0 | 0 | 2.5x10^-4^ (0.09) | 0.29 (0.13) |
|  | Polypeptides | 0 | 9.5x10^-5^ (0.11) | 0 | 0 | 9.5x10^-5^ (0.03) |  |
|  | Tetracyclines | 0.05 (100) | 0.09 (99.61) | 0.14 (100) | 0.02 (100) | 0.29 (99.88) |  |
|  | **Total** | 0.05 (100) | 0.09 (100) | 0.14 (100) | 0.02 (100) | 0.29 (100) |  |
| Parenteral | Aminoglycosides | 4.65 (12.75) | 6.2 (18.72) | 5.80 (20.45) | 9.18 (22.86) | 25.82 (18.7) | 138.03 (60.07) |
|  | Cephalosporin (3rd/4th gen) | 0 | 0.01 (0.03) | 0 | 0 | 0.01 (0.01) |  |
|  | Macrolides | 0.10 (0.27) | 0.10 (0.3) | 0 | 0 | 0.20 (0.14) |  |
|  | Penicillins | 2.84 (7.82) | 3.72 (11.23) | 4.25 (15.0) | 5.96 (14.8) | 16.77 (12.15) |  |
|  | Sulfonamides | 4.12 (11.33) | 2.61 (7.90) | 2.41 (8.5) | 4.75 (11.8) | 13.89 (10.06) |  |
|  | Tetracyclines | 24.68 (67.82) | 20.48 (61.82) | 15.91 (56.1) | 20.28 (50.5) | 81.34 (58.93) |  |
|  | **Total** | 36.38 (100) | 33.12 (100) | 28.36 (100) | 40.17 (100) | 138.03 (100) |  |
| Oral | Aminoglycosides | 0 | 0 | 0.01 (0.5) | 0.01 (0.05) | 0.03 (0.03) | 91.45 (39.80) |
|  | Fluoroquinolones | 3.81 (6.39) | 0.20 (100) | 0 | 2.00 (6.96) | 6.01 (6.57) |  |
|  | Macrolides | 25.00 (41.90) | 0 | 0 | 11.25 (39.16) | 36.25 (39.64) |  |
|  | Penicillins | 20.25 (33.94) | 0 | 2.50 (87.47) | 14.88 (51.78) | 37.63 (41.14) |  |
|  | Polypeptides | 10.04 (16.82) | 0 | 0.32 (11.33) | 0.57 (1.97) | 10.93 (11.95) |  |
|  | Sulfonamides | 0.57 (0.96) | 0 | 0 | 0 | 0.57 (0.62) |  |
|  | Tetracyclines | 0 | 0 | 0.02 (0.7) | 0.02 (0.07) | 0.04 (0.04) |  |
|  | **Total** | 59.67 (100) | 0.02 (100) | 2.86 (100) | 28.73 (100) | 91.45 (100) |  |
| All routes |  | 96.10 | 33.41 | 31.36 | 68.91 |  | 229.77 (100) |
