# Supplementary material for: Antimicrobial Use in Animals in Timor-Leste Based on Veterinary Antimicrobial Imports between 2016 and 2019
Source: Antibiotics (Basel). 2021 Apr 12;10(4):426. doi: 10.3390/antibiotics10040426 (PMC8070255; doi:10.3390/antibiotics10040426)
Supplement: Supplementary file 1 [file antibiotics-10-00426-s001.zip › Supp Material/Table S3 Type of importer (final).docx]

Table S3: Total weight of active ingredients of different classes of veterinary antimicrobials imported into Timor-Leste, by type of importer between 2016 and 2019.

|  |  | Kilogram of active ingredient (%) | | | | | r_s_ (p-value)^1^ | Kilogram for each type of importer from 2016 to 2019 (%) |
| --- | --- | --- | --- | --- | --- | --- | --- | --- |
| Type of importer | Antimicrobial class | 2016 | 2017 | 2018 | 2019 | Total from 2016 to 2019 |  |  |
| MAF | Aminoglycosides | 4.00 (16.39) | 6.00 (21.21) | 5.80 (20.43) | 9.00 (22.89) | 24.80 (20.60) |  | 120.40 (52.4) |
|  | Penicillins | 2.40 (9.83) | 3.60 (12.73) | 4.25 (14.95) | 5.60 (14.24) | 15.84 (13.16) |  |  |
|  | Sulfonamides | 0.96 (3.93) | 1.44 (5.09) | 2.35 (8.27) | 4.70 (11.95) | 9.45 (7.85) |  |  |
|  | Tetracyclines | 17.05 (69.85) | 17.25 (60.97) | 16.00 (56.34) | 20.02 (50.92) | 70.31 (58.40) |  |  |
|  | **Total** | 24.41 (100) | 28.29 (100) | 28.39 (100) | 39.31 (100) | 120.40 (100) | 1 (<0.001) |  |
| Broiler farms | Aminoglycosides | 0 | 0 | 0.01 (0.50) | 0.01 (0.23) | 0.03 (0.31) |  | 9.08 (3.95) |
|  | Fluoroquinolones | 0 | 0 | 0 | 2.00 (32.12) | 2.00 (22.02) |  |  |
|  | Penicillins | 0 | 0 | 2.50 (87.47) | 3.63 (58.22) | 6.13 (67.43) |  |  |
|  | Polypeptides | 0 | 0 | 0.32 (11.33) | 0.57 (9.10) | 0.89 (9.80) |  |  |
|  | Tetracyclines | 0 | 0 | 0.02 (0.70) | 0.02 (0.32) | 0.04 (0.44) |  |  |
|  | **Total** | 0 | 0 | 2.86 (100) | 6.23 (100) | 9.08 (100) | 0.95 (0.05) |  |
| Layer farms | Fluoroquinolones | 3.60 (6.14) | 0 | 0 | 0 | 3.60 (4.44) |  | 81.14 (35.31) |
|  | Macrolides | 25.00 (42.66) | 0 | 0 | 11.25 (50.00) | 36.25 (44.68) |  |  |
|  | Penicillins | 20.00 (34.13) | 0 | 0 | 11.25 (50.00) | 31.25 (38.51) |  |  |
|  | Polypeptides | 10.00 (17.06) | 0 | 0 | 0 | 10.00 (12.32) |  |  |
|  | Tetracyclines | 0 | 0.04 (100) | 0 | 0 | 0.04 (0.05) |  |  |
|  | **Total** | 58.60 (100) | 0.04 (100) | 0 | 22.50 (100) | 81.14 (100) | -0.40 (0.60) |  |
| Agriculture shops | Aminoglycosides | 0.56 (4.90) | 0 | 0 | 0.12 (42.55) | 0.68 (4.28) |  | 15.88 (6.91) |
|  | Fluoroquinolones | 0.20 (1.75) | 0.20 (4.81) | 0 | 0 | 0.40 (2.52) |  |  |
|  | Macrolides | 0.10 (0.87) | 0.10 (2.40) | 0 | 0 | 0.20 (1.26) |  |  |
|  | Penicillins | 0.34 (2.94) | 0 | 0 | 0.16 (57.45) | 0.50 (3.14) |  |  |
|  | Sulfonamides | 2.96 (25.9) | 0.93 (22.47) | 0 | 0 | 3.90 (24.54) |  |  |
|  | Tetracyclines | 7.28 (63.64) | 2.93 (70.31) | 0 | 0 | 10.20 (64.26) |  |  |
|  | **Total** | 11.44 (100) | 4.16 (100) | 0 | 0.28 (100) | 15.88 (100) | -0.80 (0.20) |  |
| Veterinary clinics | Aminoglycosides | 0.08 (7.78) | 0.20 (21.76) | 0 | 0.06 (10.19) | 0.34 (12.86) |  | 2.65 (1.15) |
|  | Cephalosporin (3rd/4th gen) | 0 | 0.01 (1.09) | 0 | 0 | 0.01 (0.38) |  |  |
|  | Penicillins | 0.11 (10.51) | 0.12 (13.04) | 0 | 0.20 (34.13) | 0.43 (16.21) |  |  |
|  | Polypeptides | 0 | 9.53 x 10^-5^ (0.01) | 0 | 0 | 9.53 x 10^-5^ (<0.01) |  |  |
|  | Sulfonamides | 0.44 (42.80) | 0.24 (26.08) | 0.06 (54.01) | 0.05 (8.15) | 0.79 (29.73) |  |  |
|  | Tetracyclines | 0.40 (38.91) | 0.35 (38.03) | 0.05 (45.99) | 0.28 (47.54) | 1.08 (40.82) |  |  |
|  | **Total** | 1.03 (100) | 0.92 (100) | 0.11 (100) | 0.59 (100) | 2.65 (100) | -0.80 (0.20) |  |
| Education institutions | Fluoroquinolones | 0.01 (1.59) | 0 | 0 | 0 | 0.01 (1.59) |  | 0.63 (0.27) |
|  | Penicillins | 0.25 (39.73) | 0 | 0 | 0 | 0.25 (39.73) |  |  |
|  | Polypeptides | 0.04 (6.23) | 0 | 0 | 0 | 0.04 (6.23) |  |  |
|  | Sulfonamides | 0.33 (52.45) | 0 | 0 | 0 | 0.33 (52.45) |  |  |
|  | **Total** | 0.63 (100) | 0 | 0 | 0 | 0.63 (100) | -0.77 (0.22) |  |

^1^ Spearman rank-order correlation coefficient (r_s_) and p-value assessing the strength and direction of possible a monotonic trends in the quantities of antimicrobials imported over time by class of importer
